# NVX-412, a New Oncology Drug Candidate, Induces S-Phase Arrest and DNA Damage in Cancer Cells in a p53-Independent Manner

**DOI:** 10.1371/journal.pone.0045015

**Published:** 2012-09-13

**Authors:** Alexandra Hebar, Barbara C. Rütgen, Edgar Selzer

**Affiliations:** 1 Department of Radiation Oncology, Medical University of Vienna, Vienna, Austria; 2 Institute of Immunology, Department for Pathobiology, University of Veterinary Medicine Vienna, Vienna, Austria; Duke University Medical Center, United States of America

## Abstract

The new molecular entity quinoxalinhydrazide derivative NVX-412 was identified as a promising drug candidate for the treatment of various cancer types due to its strong cytotoxic activity and relative specificity. Here, we provide first data about the mechanisms of action of NVX-412. We show that NVX-412 exerts its anti-neoplastic activity in a p53-independent manner and induces S-phase arrest and DNA damage as assessed by γH2AX staining. We suggest a bi-modal (dose-dependent) mode of action of NVX-412, being primarily cytostatic at lower and predominantly cytotoxic at higher concentrations. Based on the broad and consistent anti-neoplastic activity observed, NVX-412 holds promise as an effective drug candidate for the treatment of various cancer types, especially for hematological malignancies with highly unmet medical need.

## Introduction

**Figure 1:**
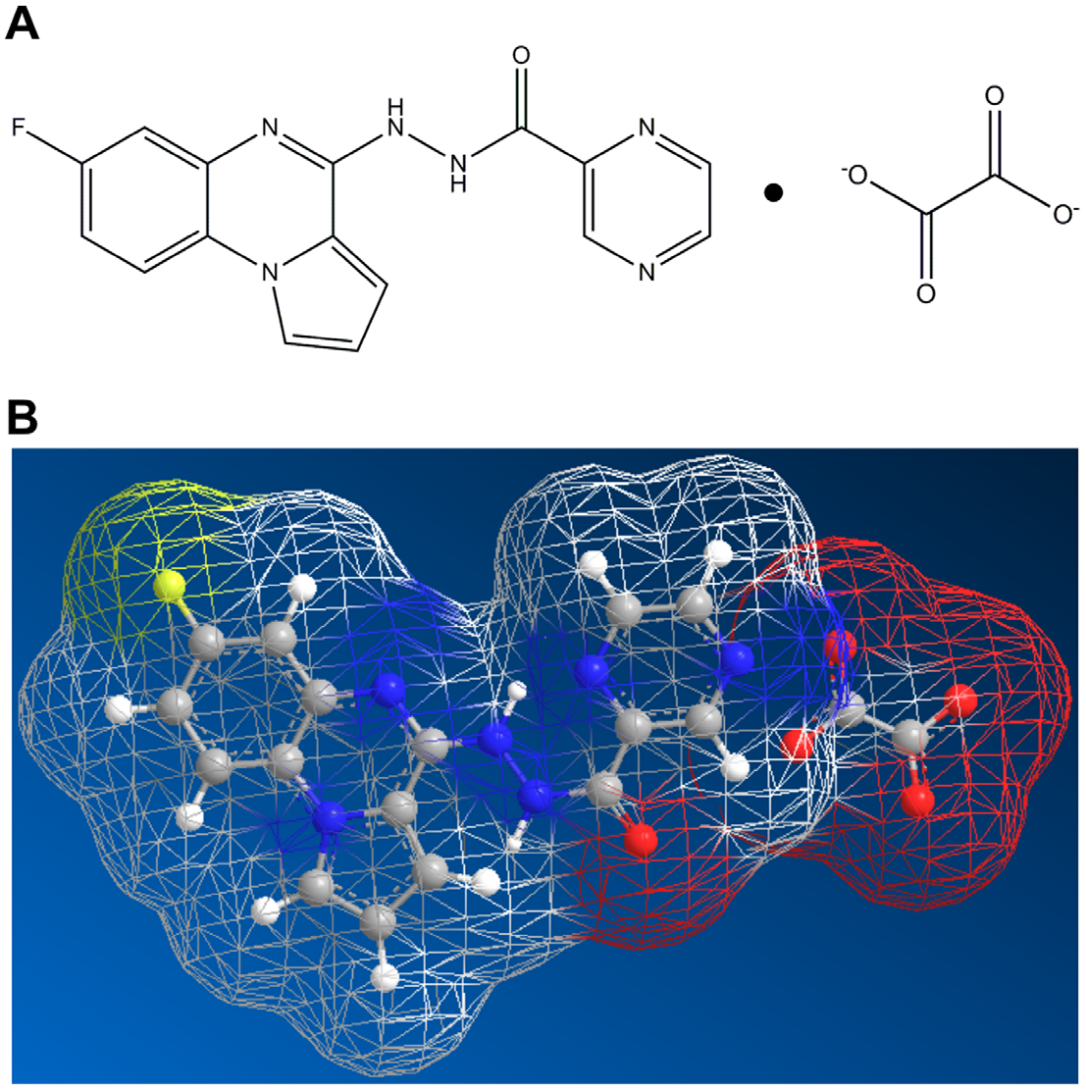
Chemical structure of NVX-412. **A:** Pyrazine-2-carboxylic acid N’-(7-fluoro-pyrrolo[1,2-α]quinoxalin-4-yl)-hydrazide-oxalic acid co-crystal; Molecular Formula: C_18_H_13_FN_6_O_5_, Molecular Weight: 412 g/mol **B:** Solvent accessible mesh model. White: carbon; yellow: fluorine; blue: nitrogen; red: oxygen. Both structures were generated with ChemBio 3D Ultra 12.0 (CambridgeSoft, MA, USA).

Cancer is one of the leading causes of death worldwide. According to the World Health Organization cancer accounts for approximately 13% of all deaths worldwide [1]. Despite extensive investment, investigation and research over decades, the currently available anti-cancer drugs lay behind expectations and therefore new, highly active, well-tolerated and ideally orally bio-available anti-cancer agents are strongly needed. Pyrazine-2-carboxylic acid N’- (7-fluoro-pyrrolo[1,2-α]quinoxalin-4-yl)-hydrazide-oxalic acid co-crystal, referred to as NVX-412 (Figure 1), is a promising drug candidate for the treatment of a number of cancer types. This new molecular entity drug candidate fulfills the criteria of Lipinskís rule of five, which gives a first hint on drug-like properties and on whether a putative drug candidate may be suitable as a medicinal product [2]. NVX-412 is a co-crystal of oxalic acid and NVX-144, its parental lead compound. NVX-144′s discovery and chemical structure was described by Grande and colleagues [3]. It belongs to the chemical class of quinoxalinhydrazides and was developed via rational drug design [3]. The fact that NVX-144 forms a co-crystal with oxalic acid is of special interest, since Aakeroy et al. have shown that co-crystals of nitrogen-containing heterocycles with carboxylic acids show advantages over the corresponding salts concerning certain physical properties favorable for pharmaceutical formulations [4]. NVX-412 confirms this notion by showing increased cytotoxic activity compared to the parental compound NVX-144 in HT-29 and HCT116 colon carcinoma cell lines with an IC_50_ that is 3 to 4-fold lower [3].

So far, the mechanism of action of NVX-412 is not known. Here, we demonstrate that NVX-412 is a promising novel anti-cancer agent that exerts its anti-neoplastic effects in a broad range of tumor cell lines of various histology. We further suggest that NVX-412 has a bi-modal mechanism of action being primarily cytostatic at lower and predominantly cytotoxic at higher concentrations. We show that NVX-412 induces S-phase arrest as well as DNA damage and a decrease in DNA replication. The mode of action of NVX-412 is independent of p53.

## Materials and Methods

### Drugs

NVX-412 (Figure 1) was obtained from Novelix Pharmaceuticals, Inc. (La Jolla, CA, USA). For *in vitro* studies, the compound was dissolved in DMSO (25 mM stock stored at −80°C) and diluted at the concentrations indicated. Nutlin-3 and (S)-(+)-Camptothecin (CPT) were purchased from Sigma-Aldrich (Vienna, AUT) and dissolved in DMSO (stocks: 3.5 mM and 5 mg/ml, respectively). All solutions were freshly prepared before use.

### NCI-60 DTP (Developmental Therapeutics Program) Human Tumor Cell Line Screen

NVX-412 was included in an anti-cancer activity screen by the National Cancer Institute (NCI) [5]. The compound was tested against 59 different human tumor cell lines, representing leukemia, melanoma and cancers of the lung, colon, brain, ovary, breast, prostate, and kidney (for a complete list of cell lines please refer to Shoemaker et al. 2006 [5]). The methodology of this *in vitro* cancer screen is described in detail on the NCI website (http://dtp.nci.nih.gov/branches/btb/ivclsp.html). Briefly, the human tumor cell lines of the cancer screening panel were grown in RPMI 1640 medium containing 5% FCS and 2 mM L-glutamine in 96-well plates at densities ranging from 5,000 to 40,000 cells/well. After 24 hours the experimental drug was added at 5 concentrations plus control and cells were incubated for additional 48 hours. For determination of the growth-inhibitory effect of the compound a sulphorhodamine B assay was performed that employs a chemical fixation step at the end of drug treatment and a subsequent staining for 10 minutes. After a washing step, absorbance was determined at 515 nm. Three different dose response parameters are then calculated: growth inhibition of 50% (GI_50_), which is the drug concentration resulting in a 50% reduction in the net protein increase, the drug concentration resulting in total growth inhibition (TGI) and the LC_50_, indicating a net loss of cells following treatment [5].

**Table 1:** NCI-60 DTP human tumor cell line screen shows strong anti-cancer activity in various cancer types.

Cancer Type	Mean IC_50_
(Number of Cell Lines Tested)	[nM]
Leukemia (6)	62
Central Nervous System Cancer (5)	167
Colon Cancer (7)	169
Non-Small Cell Lung Cancer (9)	174
Renal Cancer (8)	182
Melanoma (9)	214
Breast Cancer (5)	266
Prostate Cancer (2)	276
Ovarian Cancer (6)	377

A panel of 59 human tumor cell lines of diverse tumor entities was included in a NCI screen. This table shows the mean IC_50_ values in nM after 48 hours incubation for the various cancer types for 57 of them. Results for two cell lines were identified as outliers and were not included in the calculation of the mean IC_50_. Numbers in brackets indicate the number of cell lines tested within a certain cancer type. NCI, National Cancer Institute; DTP, Developmental Therapeutics Program.

**Figure 2:**
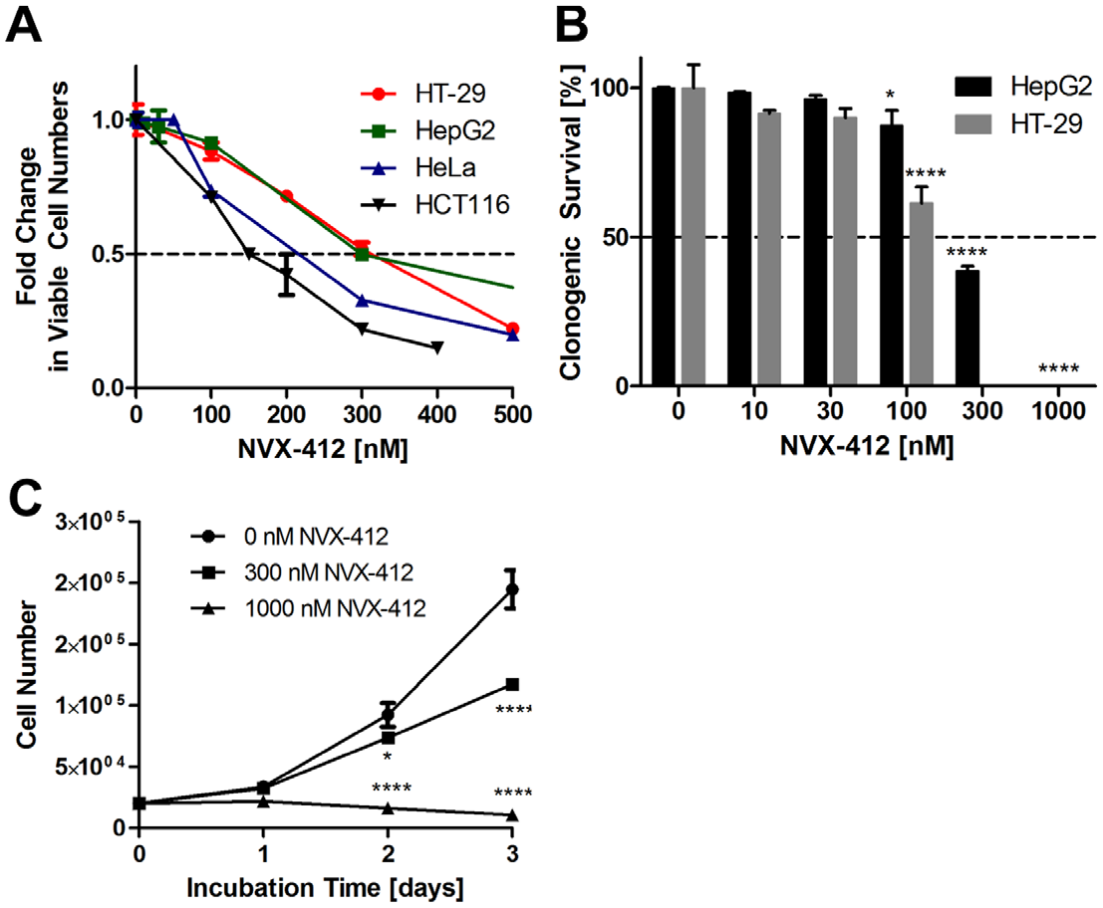
NVX-412 exerts strong anti-neoplastic and dose-dependent bi-modal activity in various tumor cell lines. A: Dose-response curves of HT-29, HepG2, HeLa and HCT116 cancer cells. Cells were incubated with the indicated concentrations of NVX-412 for 72 hours and counted with a Beckman Coulter ViCell XR. **B:** Clonogenic survival of HepG2 and HT-29 cells. HepG2 and HT-29 cells were incubated with the indicated concentrations of NVX-412 for 12 or 14 days, respectively. Clonogenic survival was significantly reduced in a dose-dependent manner. **C:** Proliferation kinetics over three days treatment with different concentrations of NVX-412 in HT-29 cells showing significantly reduced proliferation at 300 nM and a decline in cell numbers at 1 µM NVX-412 treatment. Data represent mean values (± SD) of at least 2 independent experiments. Statistical analysis was performed with GraphPad Prism 5.0. * indicates p value <0.05, **** indicates p value <0.0001 (Two-way ANOVA).

**Table 2:** IC_50_ values for 21 cell lines of various tumor entities.

Cell Line	TumorEntity	IC_50_ (nM)	Histology	Species
CLBL-1	Lymphoma	40	B-cell lymphoma	Canine
Raji	Lymphoma	60	Burkitt lymphoma	Human
SU-DHL-8	Lymphoma	80	B-cell lymphoma	Human
OSW	Lymphoma	103	T-cell lymphoma	Canine
GL-1	Leukemia	105	B-cell leukemia	Canine
Hs578T	Breast	135	Carcinoma	Human
KG-1a	Leukemia	145	AML	Human
MCF-7	Breast	150	Adenocarcinoma, pleural effusion	Human
HCT116	Colon	150	Carcinoma	Human
RKO	Colon	150	Carcinoma	Human
CL-1	Lymphoma	170	T-cell lymphoma	Canine
Ramos	Lymphoma	170	Burkitt Lymphoma	Human
KG-1	Leukemia	190	AML	Human
BV173	Leukemia	210	CML	Human
HeLa	Cervix	210	Carcinoma	Human
HL-60	Leukemia	210	AML	Human
HepG2	Liver	290	Carcinoma	Human
HT-29	Colon	300	Adenocarcinoma	Human
SU-DHL-6	Lymphoma	320	B-cell lymphoma	Human
NALM-1	Leukemia	330	CML	Human
K562	Leukemia	540	CML	Human

The table shows IC_50_ values after 72 hours incubation for 21 cell lines originating from diverse tumor entities of two species. IC_50_ values were calculated with GraphPad Prism 5.0 based on at least two independent experiments performed in duplicates.

### Cell Lines

The following cell lines were used in this study (cell lines from NCI-60 DTP human tumor cell line screen not included): The isogenic human colorectal carcinoma cell lines HCT116 p53+/+ and p53−/− and RKO p53+/+ and p53−/− were purchased from Horizon Discovery Ltd. (Cambridge, UK) and were cultured in McCoy’s 5A medium supplemented with 10% FCS, 1% Pen Strep and 2 mM L-Glutamine (whenever the p53 status of HCT116 cells is not specified p53+/+ cells were used). CLBL-1 (canine B-cell lymphoma) [6], OSW [7] and CL-1 (canine T-cell lymphoma) [8] and GL-1 (canine B-cell leukemia) [9] cell lines were kindly provided by B. C. Rütgen (University of Veterinary Medicine Vienna, Vienna, AUT), and were cultured in RPMI 1640 supplemented with 10% FCS and 1% Pen Strep. The B-cell Non-Hodgkin lymphoma cell lines SU-DHL-6 and SU-DHL-8, purchased from DSMZ (German Collection of Microorganisms and Cell Cultures, Braunschweig, GER), were cultivated in RPMI 1640 supplemented with 10% FCS and 1% Pen Strep. The same culture conditions were used for all the other lymphoma and leukemia cell lines Raji [10], [11], Ramos [10], [11], KG-1 [10], [12], [13], [14], KG-1a [14], HL-60 [10], [12], [13], BV173 [10], [11], NALM-1 [12], [13] and K562 [10], [12], [13], which were kindly provided by P. Valent (Medical University of Vienna, Vienna, AUT) and are all previously published cell lines available either from ATCC (American Type Culture Collection, Manassas, VA, USA) or DSMZ. Hs27, a normal fibroblast cell line, was provided by D. Barlow (Research Center for Molecular Medicine, Vienna, AUT) and was grown in DMEM with 10% FCS and 1% Pen Strep [15], [16]. Hs578T were grown in Minimum Essential Medium-α (MEM) supplemented with 10% FCS, 1% Pen Strep and 1% L-Glutamine and were a kind gift from T. Grunt (Medical University of Vienna, Vienna, AUT) [17]. Both cell lines, Hs27 and Hs578T, are available from ATCC. HUVEC normal human umbilical vein endothelial cells were purchased from Lonza (Walkersville, Maryland, USA) and were grown in Clonetics® EGM® BulletKit media. Human white preadipocytes (hWP) were purchased from PromoCell (Heidelberg, GER) and were cultured in preadipocyte growth medium provided by the company. The breast adenocarcinoma cell line MCF-7, the cervical carcinoma cell line HeLa, the hepatocellular carcinoma cell line HepG2 and the colorectal adenocarcinoma cell line HT-29 were obtained from ATCC and were cultured in DMEM supplemented with 10% FCS and 1% Pen Strep. If not indicated otherwise all cell culture reagents were purchased from Gibco (Grand Island, NY, USA).

**Figure 3:**
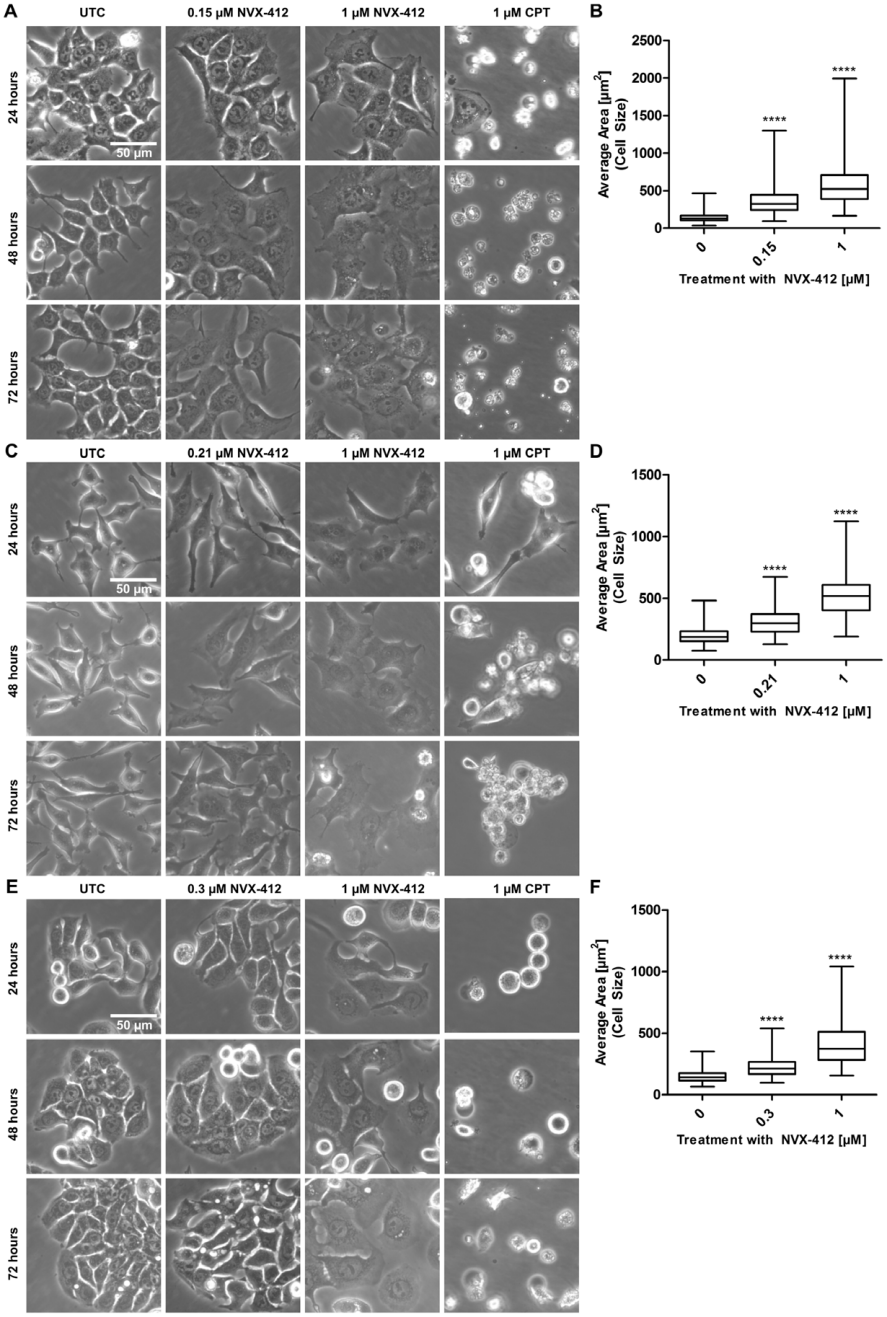
NVX-412 induces significant increase in cell sizes at IC_50_ and higher concentrations in HCT116, HeLa and HT-29 cells. HCT116, HeLa and HT-29 cells were treated for up to 72 hours as indicated. A DMSO control was performed but did not show any differences compared to UTC. After 24, 48 and 72 hours pictures were taken (**A**, **C**, **E**). Panels **B**, **D** and **F** show quantification of cell sizes after 48 hours treatment with indicated concentrations of NVX-412. Box plots represent data of cells counted within 5 different fields of view. Statistical analysis was performed with GraphPad Prism 5.0. **** indicates p value <0.0001 (Mann-Whitney U Test). **A**, **B**: HCT116. **C**, **D**: HeLa. **E**, **F**: HT-29. UTC, Untreated Control; CPT, Camptothecin.

### Cytotoxicity Assays

Cells were plated in 24-well plates. After cells were allowed to recover for 24 hours, NVX-412 was added in fresh growth medium. The maximal DMSO concentration reached in all experiments was below 0.01%. Respective control experiments at the highest DMSO concentration were performed in order to rule out DMSO-induced effects. After 72 hours incubation the proportion of viable cells was determined by cell counting with a Z1 Coulter Particle Counter (Beckman Coulter, Vienna, AUT), a ViCell XR (Beckman Coulter, Vienna, AUT) or a CASY® Cell Counter (Schärfe, Reutlingen, Germany). Cytotoxicity was expressed as IC_50_ values that were derived from the corresponding dose–response curves.

### Clonogenic Assays

For clonogenic assays, HT-29 and HepG2 cells were plated in 60 mm dishes at a density of 1000 cells per dish. After 24 hours NVX-412 was added at the indicated concentrations. After cultivation for 12–14 days (when assessable colonies were visible), colonies were fixed in 70% ethanol, stained with 0.5% Crystal Violet and counted manually.

**Figure 4:**
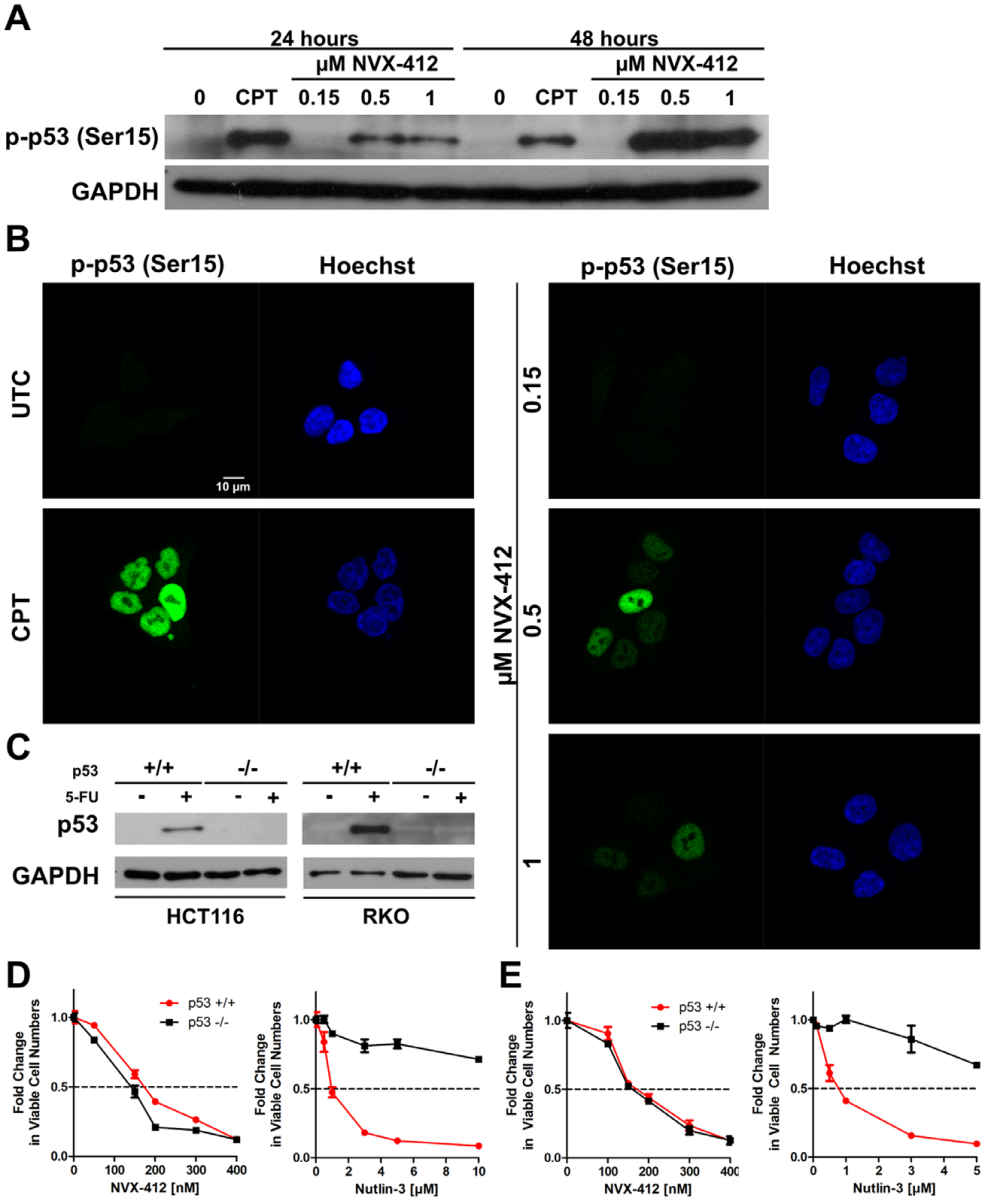
NVX-412 induces p53 phosphorylation only at higher concentrations and acts p53 status independent. A: Western Blot analysis for p-p53 (Ser15) in HCT116 cells after 24 and 48 hours incubation with 0, 0.15, 0.5 and 1 µM NVX-412 and 1 µM CPT as positive control. **B:** Corresponding immunofluorescence staining for p-p53 (Ser15) (green) of HCT116 cells after 24 hours of incubation with 0, 0.15, 0.5 and 1 µM NVX-412 and 1 µM CPT as positive control. Nuclei are stained with Hoechst 33342 (blue). **C:** Confirmation of p53 status in HCT116 p53+/+ and p53−/− cells by immunoblotting. Cells were cultured in the presence and absence of 375 µM 5-FU for 24 hours. **D**, **E**: Cell Numbers of HCT116 p53+/+ or p53−/− (**D**), RKO p53+/+ or p53−/− (**E**) cells after 72 hours incubation with NVX-412 or Nutlin-3. Cells were cultured for 72 hours with different concentrations of NVX-412 or Nutlin-3, respectively. Viable cell numbers were determined using a Beckman Coulter ViCell XR. Data represent mean values (± SD) of two independent experiments.

### Proliferation Kinetics

HT-29 cells were plated (2×10^4^ cells/well) in 24-well plates. After a recovery period of 24 hours, NVX-412 was added in fresh growth medium to final concentrations of 0, 300 and 1000 nM, respectively. After 24, 48 and 72 hours cell numbers were determined with a Z1 Coulter Particle Counter.

### Morphology Assays

To investigate potential morphological changes upon treatment with NVX-412, HCT116, HeLa and HT-29 cells were plated in 6-well plates and incubated for up to 72 hours with the indicated concentrations of NVX-412, DMSO as vehicle control and 1 µM Camptothecin (CPT) as a positive control for apoptotic morphology. Control experiments were carried out to ensure that morphological changes are not due to differences in confluency. After 24, 48 and 72 hours pictures were taken with an Olympus IX71 inverted microscope and camera system (Olympus Color View III) at 20× magnification.

### Quantification of Cell Sizes

Cell sizes were quantified using specialized imaging software (ImageJ 1.45d, US NIH, Bethesda, MD, USA). Average areas of all cells present within 5 different fields of view per sample were determined after 48 hours treatment with 0, 0.15 and 1 µM NVX-412. The non-parametric Mann-Whitney U test was used to assess the statistical significance of differences between the cell sizes, because the Kolmogorov–Smirnov test showed that the data were not normally distributed (GraphPad Prism 5.0, La Jolla, CA, USA).

**Figure 5:**
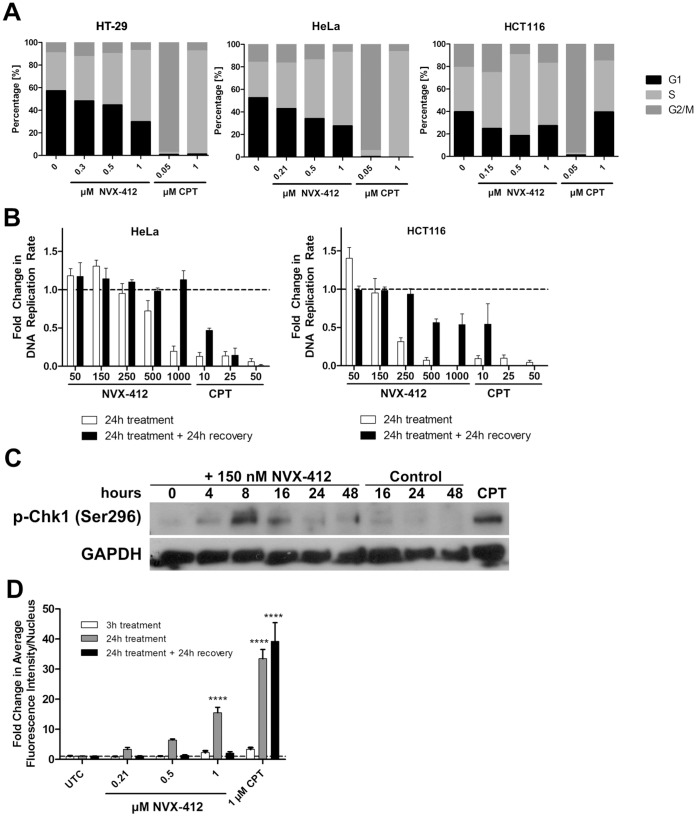
NVX-412 induces S-phase arrest and DNA damage and reduces DNA replication rate. **A:** Cell cycle analyses by flow cytometry for HT-29, HeLa and HCT116 cells treated for 24 hours as indicated with NVX-412 or CPT. Percentages of cells in G1, S and G2/M phase of the cell cycle are shown. Cells were analyzed using a BD FACScan. **B:** NVX-412 reduces DNA replication in a reversible manner in HeLa and HCT116 cells. HeLa and HCT116 cells were treated for 24 hours as indicated. Additionally, after 24 hours of treatment cells were allowed to recover for 24 hours in normal growth medium without NVX-412 or CPT. DNA replication rate was analyzed by BrdU incorporation. **C:** Western Blot for pChk1(Ser296) in HCT116 cells after 0–48 hours incubation with 150 nM NVX-412. **D:** NVX-412 induces γH2AX in HeLa cells in a reversible manner. Cells were treated for 3 and 24 hours as indicated. Additionally, after 24 hours of treatment cells were allowed to recover for 24 hours in normal growth medium without NVX-412 or CPT. Data represent fold change in average γH2AX fluorescence intensity per nucleus (± SD) as quantified from immunofluorescence stainings. UTC, Untreated Control; CPT, Camptothecin. Mean values of at least 2 independent experiments are shown. Statistical analysis was performed with GraphPad Prism 5.0. **** indicates p value <0.0001 (Two-way ANOVA).

### Western Blot

Treated cells were directly lysed in NP40 cell lysis buffer (Invitrogen, Grand Island, NY, USA) containing protease and phosphatase inhibitors. Protein concentrations were measured colorimetrically (D_C_ Protein Assay, Bio-Rad Laboratories, Hercules, CA, USA). Proteins were separated by SDS - polyacrylamide gel electrophoresis and blotted onto nitrocellulose membranes (Whatman™, Vienna, AUT). Equal loading was checked by Ponceau S (SERVA, Heidelberg, GER) staining. Bound antigen was visualized with the enhanced chemiluminescence detection system (Roche Diagnostics, Indianapolis, IN, USA). These procedures were performed according to the manufacturer’s protocols. Antibodies specific for the following proteins of interest were used: pChk1 (Ser296), p-p53 (Ser15), β-Tubulin and GAPDH were obtained from Cell Signaling Technology (Danvers, MA, USA) at 1∶1000 dilution. Secondary antibodies were peroxidase-tagged goat anti-rabbit or anti-mouse IgGs (Cell Signaling Technology) (1∶2000).

### Immunofluorescence Staining of p-p53 (Ser15) and γH2AX (Ser139)

HCT116 and HeLa cells were seeded on glass cover slips in 6-well plates. 24 hours after plating, HCT116 cells were incubated with 0.15, 0.5 and 1 µM NVX-412 and 1 µM CPT for 24 hours for staining of p-p53. For investigating γH2AX, HeLa cells were incubated with 0.21, 0.5 and 1 µM NVX-412 and 1 µM CPT for 3 or 24 hours. Additionally, cells were allowed to recover for 24 hours in normal growth medium after 24 hours of incubation with NVX-412 or CPT (wash-out experiment). After the respective treatments, cells were washed and fixed with 4% methanol-free formaldehyde (Polysciences Inc. Warrington, PA, USA) for 15 minutes at room temperature and then permeabilized with 0.2% Triton X-100/PBS for 2 minutes at room temperature. After blocking with goat serum (5%) in 1% BSA/0.3% Triton X-100/PBS, the respective primary antibody was added and cells were incubated at 4°C overnight. Antibodies used were: mouse p-p53 (Ser15) (Cell Signaling Technology, Danvers, MA, USA) at 1∶100 dilution and mouse p-H2AX (Ser139) (Millipore, Billerica, MA, USA) at 1∶1000 dilution in 1% BSA/PBS, respectively. Cells were then washed 3 times for 10 minutes with 0.25% BSA/0.1% Triton X-100/PBS and incubated with the Alexa Fluor 488 conjugated secondary anti-mouse antibody (1∶1000 in 0.25% BSA/0.1% Triton X-100/PBS) for 1 hour at room temperature in the dark. Cells were washed 3 times for 10 minutes with 0.05% Tween-20/PBS containing Hoechst 33342 (Sigma Aldrich, Vienna, AUT) and mounted with mounting medium (Fluoprep, bioMérieux, Marcy l'Etoile, FRA). Fluorescence was immediately recorded on a Zeiss LSM700 laser scanning microscope.

### Quantification of γH2AX

The common procedure for assessing γH2AX induction is to count γH2AX foci. Since with NVX-412 and the positive control CPT γH2AX activation was that strong, no distinguishable and countable foci were seen. Therefore, the average γH2AX fluorescence intensity per nucleus was determined. To this end, γH2AX intensities within a certain field were measured using Quantity One –4.6.9 (Basic freeware version, Bio-Rad Laboratories, Hercules, CA, USA) and were divided by the number of nuclei in this field.

### Determination of p53 Status Dependency

The isogenic colon carcinoma cells lines HCT116 p53+/+ or p53−/− and RKO p53+/+ or p53−/− were cultured in 12-well plates. After a recovery period of 24 hours, NVX-412 or Nutlin-3 was added in fresh growth medium at the indicated concentrations. Nutlin-3, a MDM2 antagonist and hence p53 pathway activator was used to prove the differential biological effects of the p53 status. After 24 or 72 hours cell numbers were determined with a Beckman Coulter ViCell XR.

### DNA Replication Rate

HeLa and HCT116 cells were plated in black 96-well plates. After cells were allowed to recover for 24 hours NVX-412 or CPT was added in fresh growth medium. After 24 hours of incubation a chemiluminescent BrdU Incorporation ELISA (Roche Applied Science, Penzberg, Germany) was performed with half of the plates according to the manufacturer’s protocol. Cells in the remaining plates were allowed to recover for 24 hours in normal growth medium before DNA replication was measured (wash-out experiment). To check whether a possible decline in DNA replication rate is not simply due to lower cell numbers because of cell death induction, the proportion of viable cells was determined in parallel by a MTT-based cytotoxicity assay according to the manufacturer’s protocol (EZ4U, Biomedica, Vienna, Austria). Colorimetric (at 492 and 620 nm) and chemiluminescent measurements were performed using a FLUOstar OPTIMA (BMG Labtech, Ortenberg, Germany).

### Flow Cytometric Cell Cycle Analyses

HT-29, HeLa and HCT116 cells were plated in 6-well plates. After cells were allowed to recover for 24 hours, NVX-412 was added in fresh growth medium at the respective IC_50_ concentration and 0.5 and 1 µM. CPT was used at 50 nM and 1 µM. To analyze the cell cycle distribution, cells were collected after 24 hours of incubation and washed with PBS. Cells were fixed in 70% ethanol for at least 2 hours. For analysis, cells were transferred into PBS, incubated with RNAse A (0.04 µg/ml final concentration) for 30 minutes at 37°C, treated with 40 µg/µl propidium iodide for 30 min at 4°C and then analyzed by flow cytometry using BD FACScan (Becton, Dickinson and Company, Franklin Lakes, NJ, USA). The resulting DNA histograms were quantified using ModFit LT (Verity Software House, Topsham, ME, USA).

### Statistical Analyses

If not indicated otherwise, two-way ANOVA (Analysis of Variance; GraphPad Prism 5.0) was used to assess the statistical significance of differences between the data.

## Results

### NVX-412 Exerts Strong Anti-neoplastic Activity

To obtain an independent overview of the range of activity of NVX-412 (Figure 1) against a panel of well-described cancer cell lines, the drug candidate was included in an anti-cancer activity screen conducted by the NCI against 59 different human tumor cell lines originating from various cancer types. This screen revealed a very strong and broad anti-cancer activity of NVX-412 in tumor cell lines of all cancer types in the low nanomolar range with a mean IC_50_ of about 200 nM (Table 1). Leukemic cell lines were most sensitive with a mean IC_50_ of 62 nM. In addition to the NCI-60 DTP Human Tumor Cell Line Screen (see Table 2), additional cell lines derived from various tumor entities and two different species including human and canine cells and also normal non-cancer cells were tested. In agreement with the data obtained in the NCI screen, these data showed anti-cancer activity in all cell lines tested all across a variety of tumor types. Figure 2A shows dose-response curves for HT-29 colon adenocarcinoma, HepG2 hepatocellular carcinoma, HeLa cervical carcinoma and HCT116 colon carcinoma cells, which are cell lines used for all further experiments in this study. Normal human endothelial cells (HUVEC) and human white preadipocytes (hWP) displayed a reduced sensitivity compared to cancer cell lines. For HUVEC cells the IC_50_ was determined at 2.0 µM and for human white preadipocytes hWP at 1.0 µM, respectively.

### NVX-412 Shows Bi-modal Activity and Induces Morphological Changes

To better understand the NVX-412-induced effects, cell growth, cell survival and cell death experiments were performed. Clonogenic survival assays [18] were performed with HepG2 and HT-29 cells. The cells were incubated with different concentrations of NVX-412 for 12 or 14 days, respectively. As can be seen in Figure 2B, NVX-412 reduced the ability of HepG2 and HT-29 cells to form colonies in a dose-dependent manner. Of note, the concentration of NVX-412 to achieve half-maximal effects determined by the clonogenic assay is similar to the concentration as determined by the 3-day proliferation assay. Proliferation kinetics was determined over 3 days of treatment with HT-29 colon carcinoma cells (Figure 2C). At the IC_50_ (300 nM), proliferation of cells was significantly decreased after 2 days compared to the untreated control cells. At concentrations above the IC_50_ (at 1 µM), cell numbers started to decline below the numbers of cells seeded at the beginning of the experiment, suggesting a direct induction of cell death as well as cell cycle arrest. Furthermore, the morphology of cells exposed to NVX-412 at either the IC_50_ or higher concentrations (1 µM) was investigated in HCT116, HeLa and HT-29 cells (Figures 3A, C, E). Treatment with NVX-412 at the IC_50_ led to changes in the morphological appearance of all three investigated cell types within 24 hours; the cells appeared larger than untreated cells. DMSO alone as vehicle control did not change the morphological appearance (DMSO control not shown). To investigate this interesting phenomenon in more detail, cell sizes were quantified after 48 hours treatment with NVX-412. A highly significant increase in cell sizes was seen for all three tested cell lines (Figure 3B, D, F). Over the next 48 hours a decrease of cell densities could be observed for HCT116, HeLa and HT-29 cells. However, this picture changed dramatically when higher concentrations of NVX-412 were applied. Again, cells displayed an altered morphological appearance and enlarged size compared to untreated cells (Figure 3), but already after 48 hours the number of detached and dead cells increased (data not shown), similar to the treatment with the cell death inducer CPT.

### NVX-412 Acts p53 Status Independent

To investigate a possible p53 status dependency of NVX-412, the phosphorylation status of p53 at Ser15 was determined [19], [20]. Immunofluorescence stainings for p53 phosphorylated at Ser15 were performed in HCT116 cells after 24 hours of incubation with different concentrations of NVX-412 (Figure 4B). As expected, cells treated with 1 µM CPT were positive for nuclear staining of p-p53 (Ser15), whereas untreated cells were clearly negative. Incubation with the IC_50_ concentration of NVX-412 did not lead to p-p53 (Ser15) staining, while cells treated with higher concentrations (0.5 and 1 µM) showed a positive staining. This correlates with Western Blot analysis that was performed under the same conditions and at the same time points after 24 and 48 hours (Figure 4A). Again, untreated cells and cells treated below or at the IC_50_ were negative for phosphorylated p53, whereas the control experiment with 1 µM CPT and cells treated with NVX-412 at concentrations above the IC_50_ showed a strong induction.

To further investigate a potential p53 status dependency of the activity of NVX-412 two different isogenic cell line models only differing in their p53 status were investigated; HCT116 cells with a p53+/+ or p53−/− phenotype, and RKO cells with a p53+/+ or p53−/− phenotype. The p53 status of the cells was confirmed by immunoblotting (Figure 4C). In Figure 4D and 4E the dose-response curves for NVX-412 and the control Nutlin-3 in p53+/+ and p53−/− cells are shown. It could be demonstrated that in both cell lines, HCT116 and RKO, the p53 status does not influence the sensitivity to NVX-412; the IC50 values for the isogenic cell lines were comparable. In contrast to that the response to Nutlin-3 showed a clear p53 status dependency, with p53+/+ cells being much more sensitive than p53−/− cells.

### NVX-412 Induces S-phase Arrest, Increases Levels of DNA Damage Markers and Reduces DNA Replication

Based on the results described above, we sought to gain more insight into cell cycle effects of NVX-412. We therefore performed flow cytometric cell cycle analyses in HT-29, HeLa and HCT116 cells. In all three cell lines investigated a dose-dependent increase in the fraction of S-phase cells was observed after 24 hours of incubation with NVX-412 (Figure 5A). Already after 24 hours of treatment with NVX-412 at IC_50_ concentrations an increase in the relative numbers of S-phase cells was observed that became even more pronounced with higher concentrations. Additionally, an increase in the sub-G0/G1 cell population, which is characteristic for apoptotic cells, was observed at higher concentrations of NVX-412 and also of CPT (data not shown). CPT served as positive control for G2/M- or S-phase arrest. In HT-29 and HeLa cells CPT induced G2/M arrest at 50 nM and S-phase arrest at 1 µM. In HCT116 cells 50 nM CPT induced also G2/M-phase arrest, whereas at a concentration of 1 µM most of the cells were already dead (not shown in the figure). The cell cycle delay observed by FACS analysis is compatible with the results from BrdU incorporation ELISAs that showed a reduction of DNA replication upon NVX-412 treatment in HeLa and HCT116 cells (Figure 5B). Again, CPT was used as a positive control for reduction of DNA replication rate. Interestingly, when cells were allowed to recover for 24 hours in normal growth medium after the 24-hour treatment, the DNA replication recovered and the rate increased to normal levels both in HeLa cells and in HCT116 cells. After CPT treatment a recovery was only observed at the lowest concentration tested. To study a possible DNA damage inducing effect of NVX-412, changes in the Ser296 phosphorylation status of Chk1 were investigated. Phosphorylation was found to be increased after 4 hours of NVX-412 treatment, which is indicative for a DNA damage effect (Figure 5C). To follow up on this observation, the induction of γH2AX foci was determined by immunofluorescence staining in HeLa cells (Figure 5D). γH2AX levels were found to be elevated after a 24 hour treatment in a dose-dependent manner. In line with the ability of the cells to re-establish their normal DNA replication rate after a 24 hours recovery period (Figure 5B), γH2AX levels decreased to nearly basal levels within 24 hours after withdrawal of NVX-412 (Figure 5D, black bars). The effect of 1 µM CPT that was used as positive control for γH2AX induction was not reversible.

## Discussion

We investigated the anti-neoplastic activity of NVX-412, a new drug candidate. In a comprehensive screen performed by the NCI, NVX-412 was found to exert strong anti-cancer activity in the submicromolar range with an average IC_50_ of 200 nM for all cell lines combined. Additional data collected from 21 cancer cell lines underlined the potent anti-cancer activity of NVX-412. Our initial findings provide first evidence that NVX-412 is an interesting research – stage drug candidate that may hold promise as a novel therapeutic, particularly against hematological malignancies.

Clonogenic survival assays demonstrated that NVX-412 significantly reduced or completely blocked the capacity of HepG2 and HT-29 cells to form colonies. These results nicely confirmed results from short-term proliferation experiments. Proliferation kinetics of HT-29 cells over three days demonstrated that at concentrations of NVX-412 below or at the IC_50_ primarily the proliferation rate was reduced, whereas at higher concentrations also cell numbers declined, suggesting induction of cell death. Analysis of cellular morphology after treatment with NVX-412 underlined these findings. In contrast to the effects at higher concentrations, at which after 48 hours treatment detached dead cells were detectable in high numbers, hardly any signs of cell death were observed at concentrations below the IC_50_. These observations suggest that NVX-412 exerts its anti-neoplastic effect via different mechanisms showing a possible bi-modal concentration dependence. Of note, the morphological appearance of the cell lines was changed by NVX-412 within 24 hours; a statistically significant enlargement of cell sizes was observed after 48 hours. Enlarged cell size may be an indication for the induction of senescence, a state of permanent cell cycle arrest [21]; however, β-galactosidase staining, a standard assay for investigating cellular senescence, was negative in all cell lines tested (data not shown). An increase in cell size seems to be associated with the S-phase arrest induced by NVX-412. It has been shown by others that cells continuously increase their size from G1 up to the entry into the S-phase [22], [23], [24], which fits our observations. As mentioned above, in flow cytometric cell cycle analyses performed with three different cancer cell lines, a clear and dose-dependent increase in the fraction of S-phase cells was observed; additionally a sub G0/G1 cell population was observed at higher concentrations of NVX-412, indicating apoptotic cell death. Taken together, our results demonstrate that at lower concentrations, NVX-412 primarily triggers cell cycle arrest whereas at higher concentrations NVX-412 additionally induces cell death in a direct manner. To investigate whether the observed increase in S-phase cells is due to S-phase arrest rather than enhanced proliferation and DNA synthesis we performed BrdU incorporation ELISAs investigating the DNA replication rate of HeLa and HCT116 cells. These studies showed that after 24 hours of treatment with NVX-412 the DNA replication rate decreased in both cell lines. Interestingly, this effect was reversible in wash-out experiments. DNA replication rates increased back to nearly normal levels after 24 hours without the drug. In contrast, the effect of CPT on proliferation was not reversible.

Based on the observations that NVX-412 induces S-phase arrest and also reduces DNA replication rate we investigated a possible DNA damage inducing effect of NVX-412. Therefore we performed Western Blot analyses of Chk1, which is phosphorylated at several residues following replication stress and DNA damage and plays an important role in the DNA damage checkpoint control [25]. In fact, Chk1 Ser296 phosphorylation, which is important for the spread of Chk1 signals [25], was increased rapidly within 4 hours after NVX-412 treatment. This prompted us to study a possible DNA damage inducing effect of NVX-412 by investigating phosphorylation of H2AX as a marker for DNA damage. The histone variant H2AX is phosphorylated and forms nuclear foci at sites of DNA damage [26]. γH2AX serves as a molecular sensor for double strand breaks and was shown to be involved in the recognition of several types of DNA damage such as stalling of replication forks or abrogation of the S-phase checkpoint [27], [28]. We found that NVX-412 clearly induces DNA damage in a time- and dose-dependent manner as assessed by quantification of γH2AX immunofluorescence stainings. Only a slight induction of γH2AX was observed after 3 hours both with NVX-412 and the positive control CPT. After 24 hours of treatment γH2AX staining increased up to 15-fold (p-value <0.0001) at the highest concentration of NVX-412 tested. Furthermore it could be shown that γH2AX levels dropped to basal levels after a 24 hours recovery period in the absence of NVX-412, but not after CPT treatment. At the moment, we can only speculate about reasons for the reversibility of the NVX-412 effect. In contrast to CPT, which induces DNA strand breaks via the formation and stabilization of topoisomerase I cleavage complexes [29], [30], one could assume that NVX-412 does not directly damage the DNA, but interferes with mechanisms important for sustaining DNA integrity during DNA replication like the DNA damage response and DNA repair pathways. Once these mechanisms are not working properly the endogenously occurring DNA damage accumulates to a level at which DNA replication is slowed and S-phase arrest is induced to allow for repair [31]. Upon drug withdrawal these pathways could resume their activity, leading to a decrease of DNA damage markers and finally, DNA replication recommences. However, at this point this hypothesis is highly speculative, several other explanations may be possible, and additional studies investigating effects of NVX-412 on DNA repair will be needed for a more thorough understanding of the underlying mechanisms.

We next investigated the involvement of p53 in the anti-neoplastic effects of NVX-412. The phosphorylation status of p53 at Ser15 was assessed by immunofluorescence staining and Western Blots, respectively. p53 is an important tumor suppressor that is known to play a key role in mediating cellular stress responses. p53 exerts its effects by inducing or repressing numerous genes that are involved in cell cycle arrest, senescence, apoptosis and DNA repair [19], [20]. This makes p53 an important player in the anti-tumor response of stress-inducing chemotherapeutic agents. We could show that NVX-412 induces phosphorylation of p53 in cells treated with concentrations above the IC_50_. These findings suggest that the anti-neoplastic effect of NVX-412 at lower concentrations is not dependent on p53 activation. Importantly, mutations and alterations in p53 are the most frequent genetic events observed in human cancers, with varying frequencies depending on the cancer type [19], [32]. Many anti-cancer compounds are active in a p53-dependent manner, which means that their effectiveness is impaired in tumors with dysfunctional p53. This effect may lead to cellular resistance to a number of chemotherapeutic agents [33], [34]. Therefore we were interested in a possible p53 status dependency of the activity of NVX-412 in more detail. We investigated two different isogenic cell line models; HCT116 p53+/+ or p53−/− cells and RKO p53+/+ or p53−/− cells. We could show that in both cell lines, HCT116 and RKO, the p53 status does not influence the sensitivity to NVX-412; the IC50 values for the isogenic cell lines were comparable. Nutlin-3, a MDM2 (mouse double minute 2) antagonist and hence p53 pathway activator was used to demonstrate the differential biological effects of the p53 status. Only cells expressing functional p53 are sensitive to this compound [35], [36]. As expected, treatment with Nutlin-3 showed a clear p53 status dependency, with p53+/+ cells being much more sensitive than p53−/− cells. These results allow us to conclude that the anti-neoplastic activity of NVX-412 is independent of the p53 status and could therefore be employed in a broad spectrum of tumors.

Here, we demonstrated that NVX-412 induces S-phase arrest in various cancer cell lines. It also decreases DNA replication and elevates levels of γH2AX, a marker for DNA damage; effects that were shown to be reversible at all concentrations tested. We propose that NVX-412 exerts primarily cytostatic activity at lower and cytotoxic activity at higher concentrations suggesting a bi-modal mechanism of action. Taken together, the data presented identify NVX-412, a new molecular entity compound, as a promising drug candidate for the treatment of various cancer types. Our observations indicate that NVX-412 possesses a clinically useful dose-response relationship independent of the p53 status as well as a preferential effect on cancer over normal cells.
